# Recent pharmacological advances on genistein in clinical trials

**DOI:** 10.17179/excli2020-2675

**Published:** 2020-08-05

**Authors:** Shimy Mathew, Cijo George Vazhappilly

**Affiliations:** 1 Department of Biotechnology, American University of Ras Al Khaimah, Ras Al Khaimah, United Arab Emirates

## ⁯

***Dear Editor,***

Genistein [4′,5,7-trihydroxyisoflavone or 5,7-dihydroxy-3-(4-hydroxyphenyl) chromen-4-one] is a plant-derived, hydrolyzed aglycone form of isoflavonoid, genistin (a glycoside) (Nayeem et al., 2019[16]; Sansai et al., 2020[20]). Genistein is mainly found in leguminous plants, especially in soybeans along with other important isoflavones, daidzein and glycitein (Kalaiselvan et al., 2010[12]). Other sources of genistein include broad beans, chickpeas, vegetables, fruits, nuts, soy-based foods and genistein supplements. It has also been reported that unfermented soybeans contain more genistein than the fermented ones (Kuligowski et al., 2017[13]; Li and Zhang, 2017[14]). Further, genistein can be metabolically synthesized by introducing the* IFS* gene (isoflavone synthase gene) into yeast cells and rice lines, which results in an increased genistein content in rice (up to 30 folds) (Spagnuolo et al., 2015[23]).

Plant flavonoids, especially isoflavonoids, have shown promising pharmacological properties to ameliorate diseases including cancer (George et al., 2016[9], 2017[8]). Genistein exhibits various pharmacological properties including antioxidant properties, mainly by increasing the activity of antioxidant enzymes. It also has numerous clinical implications in the treatment and prevention of diseases like diabetes, cardiovascular diseases, cancer, and osteoporosis (Kalaiselvan et al., 2010[12]). Genistein has been reported in exhibiting anti-angiogenesis property by regulating vascular endothelial growth factor 165 and matrix metalloprotease-2 and 9 in human bladder cancer cell lines (Su et al., 2005[24]). Due to genistein's high structural similarity to estradiol, the binding capacity of genistein to the estrogen receptor is notable and thus, genistein is mainly studied in postmenopausal women. Here, we have summarized the most recent findings on the pharmacological activities of genistein in clinical trials, see Table 1(Tab. 1) (References in Table 1: Amanat et al., 2018[1]; Arcoraci et al., 2017[2]; Bilir et al., 2017[3]; Braxas et al., 2019[4]; Cho et al., 2019[5]; De Gregorio et al., 2017[6]; Dong et al., 2020[7]; Hashem et al., 2018[10]; Jochum et al., 2017[11]; Li and Zhang, 2017[14]; Lu et al., 2018[15]; Nayeem et al., 2019[16]; Orsatti et al., 2018[17]; Perez-Alonso et al., 2017[18]; Pintova et al., 2019[19]; Schneider et al., 2019[21]; Silva et al., 2017[22]; Zhang et al., 2019;[25]). 

## Acknowledgement

The authors thank the American University of Ras Al Khaimah for the support and facilities provided. The authors also thank Ms. Gulbahor Amirova, Instructor of English, the American University of Ras Al Khaimah, for proof-reading the article.

## Conflict of interest

The authors declare no conflict of interest.

## Figures and Tables

**Table 1:**
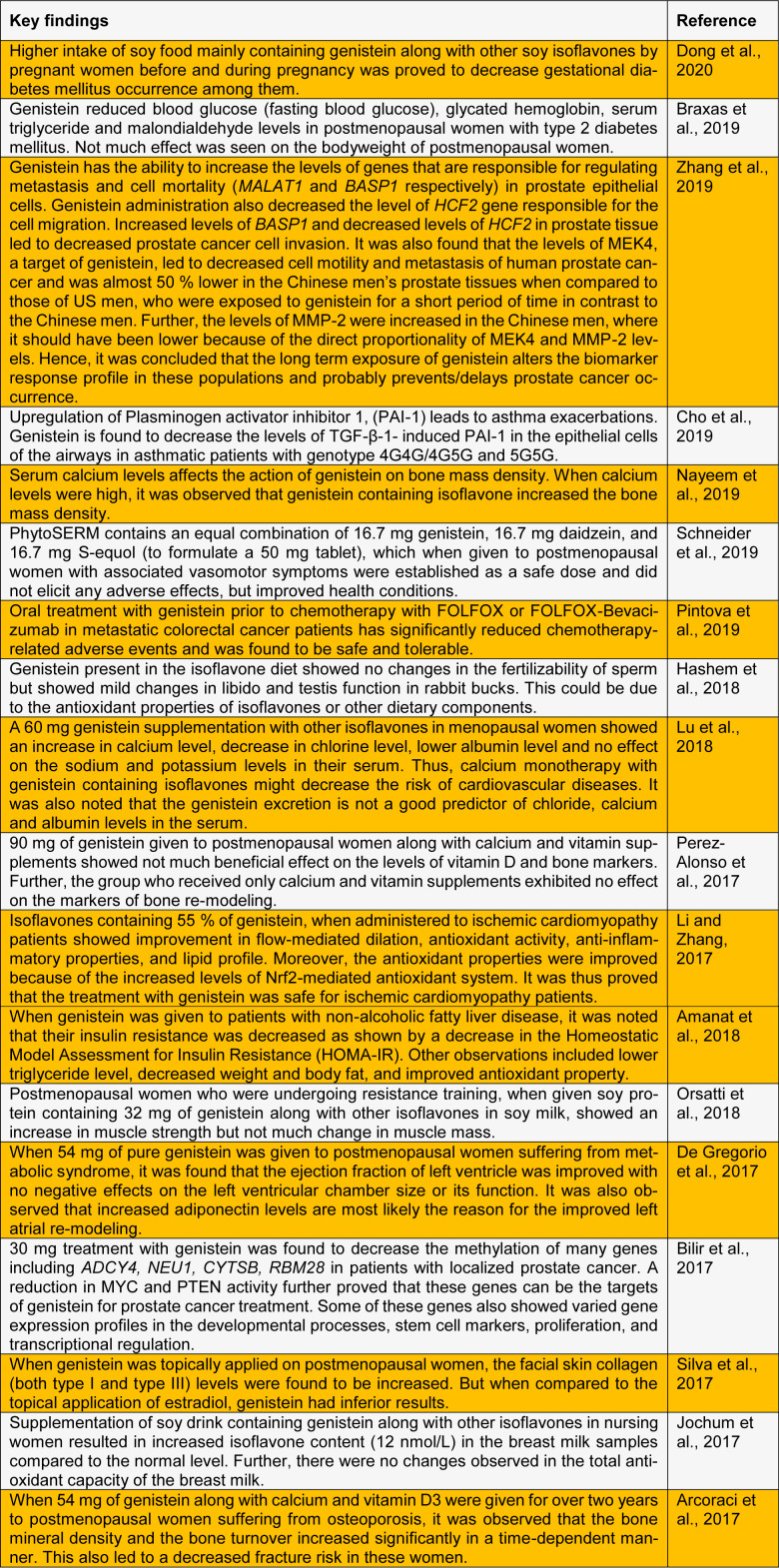
Recent findings on the pharmacological activities of genistein in clinical trials
